# Serum CYFRA 21.1 Level Predicts Disease Course in Thyroid Cancer with Distant Metastasis

**DOI:** 10.3390/cancers13040811

**Published:** 2021-02-15

**Authors:** Chaiho Jeong, Jeongmin Lee, Hyukjin Yoon, Jeonghoon Ha, Min-Hee Kim, Ja-Seong Bae, Chan-Kwon Jung, Jeong-Soo Kim, Moo-Il Kang, Dong-Jun Lim

**Affiliations:** 1 Department of Internal Medicine, Division of Endocrinology and Metabolism, Seoul St. Mary’s Hospital, College of Medicine, The Catholic University of Korea, Seoul 06591, Korea; cerbere@naver.com (C.J.); hajhoon@catholic.ac.kr (J.H.); mikang@catholic.ac.kr (M.-I.K.); 2 Department of Internal Medicine, Division of Endocrinology and Metabolism, Eunpyeong St. Mary’s Hospital, College of Medicine, The Catholic University of Korea, Seoul 06591, Korea; 082mdk45@catholic.ac.kr (J.L.); benedict@catholic.ac.kr (M.-H.K.); 3 Department of Nuclear Medicine, Seoul St. Mary’s Hospital, College of Medicine, The Catholic University of Korea, Seoul 06591, Korea; aksasin@catholic.ac.kr; 4 Department of General Surgery, Seoul St. Mary’s Hospital, College of Medicine, The Catholic University of Korea, Seoul 06591, Korea; drbae@catholic.ac.kr (J.-S.B.); btskim@catholic.ac.kr (J.-S.K.); 5 Department of Hospital Pathology, Seoul St. Mary’s Hospital, College of Medicine, The Catholic University of Korea, Seoul 06591, Korea; ckjung@catholic.ac.kr

**Keywords:** Cyfra 21.1, thyroid neoplasms, biomarkers, thyroglobulin

## Abstract

**Simple Summary:**

The role of serum Cyfra 21.1 as a biomarker in thyroid cancer has yet to be validated. This study investigated the diagnostic or prognostic role of serum Cyfra 21.1 in thyroid cancer. In the present analysis, we found that serum Cyfra 21.1 was increased in thyroid cancer with distant metastasis compared with thyroid cancer without metastasis. Furthermore, in progressive disease when thyroglobulin was undetectable or thyroglobulin monitoring was useless because of thyroglobulin antibody, serial follow-up based on serum Cyfra 21.1 levels might be used as an alternative biomarker for disease monitoring.

**Abstract:**

Background: Serum Cyfra 21.1, the soluble fragment of CK19, has been used as a prognostic tumor marker in various cancers, indicating poor tumor differentiation and increased metastasis. Methods: We analyzed the serum Cyfra 21.1 level in 51 consecutive patients with thyroid cancer manifesting distant metastasis treated with prior total thyroidectomy. Serum Cyfra 21.1 levels of 26 thyroid cancer patients without metastasis and 50 healthy individuals were used for comparison. Results: Higher serum Cyfra 21.1 levels were detected in thyroid cancer patients with distant metastasis compared with healthy subjects and thyroid cancer patients without metastasis (*p* = 0.012). Serum Cyfra 21.1 levels were significantly increased in patients with positive BRAF V600E mutation (*p* = 0.019), undergoing Tyrosine Kinase Inhibitor (TKI) therapy (*p* = 0.008), with radioiodine-refractory status (*p* = 0.047), and in disease progression compared with those manifesting stable disease (*p* = 0.007). In progressive disease with undetectable or unmonitored thyroglobulin because of thyroglobulin antibody, serum Cyfra 21.1 was useful as a biomarker for follow-up of disease course. Conclusion: Serum Cyfra 21.1 in thyroid cancer patients might represent an alternative biomarker predicting tumor progression, especially in cases not associated with serum Tg levels.

## 1. Introduction

Cytokeratin 19 (CK19) is the lowest molecular weight (40 kDa) member belonging to the cytokeratin family of proteins and contributes to the structural rigidity in simple epithelia and basal layer of most glandular and squamous epithelia in normal tissue [1]. CK19 immunostaining is used as a screening marker for neoplasm of epithelial origin because of its wide distribution in many epithelial tissues [2,3,4]. CK19 is highly expressed in differentiated thyroid cancer (DTC), but not in benign thyroid tumors [5,6,7]. Cyfra 21.1 is a soluble fragment of CK19. The prognostic value of serum Cyfra 21.1 has been shown in cancers of breast, lung, bladder, and pancreas [8,9,10,11]. Previous studies have shown that an increased serum Cyfra 21.1 level reflects poorer tumor differentiation and additional metastasis in hepatocellular carcinoma [12] and in lung cancer [13]. However, the role of serum Cyfra 21.1 as a biomarker in thyroid cancer has yet to be validated.

Few studies have evaluated the role of serum Cyfra 21.1 level in thyroid cancer. Serum Cyfra 21.1 levels did not show significant differences in patients with either benign or malignant thyroid tumors [14]. Furthermore, the levels did not differ between DTC groups without distant metastasis compared with those diagnosed with pulmonary metastasis [15]. However, a single study reported that a high serum Cyfra 21.1 level reflected the risk of distant spread of disease, suggesting a potential role as a tool to predict survival in DTC patients [16]. As mentioned above, the role of Cyfra 21.1 in thyroid cancer patients has yet to be established unequivocally.

The aim of this study was to investigate the diagnostic or prognostic role of serum Cyfra 21.1 in thyroid cancer patients. Our study also elucidated the value of serum Cyfra 21.1 as a biomarker in thyroid cancer patients with metastasis after thyroidectomy.

## 2. Materials and Methods

### 2.1. Study Population

We analyzed the serum Cyfra 21.1 level in 51 consecutive patients with thyroid cancer manifesting distant metastasis (DM-TC) and treated between 2011 and 2020 in one tertiary referral hospital. All metastatic patients underwent prior total thyroidectomy. For comparison, the serum Cyfra 21.1 levels of 26 newly diagnosed DTC patients without clinically evident lymph node or distant metastasis before surgery, non-metastatic thyroid cancer (NM-TC) and those of 50 healthy controls (Healthy) with normal thyroid gland were used. The data of serum Cyfra 21.1 levels of healthy subjects were recruited in their routine health screening program. This study complied with the ethical standards of the Helsinki Declaration and was approved by the Catholic University of Korea, Catholic Medical Center, Seoul St. Mary’s hospital Institutional Review Board (IRB approval No. KC20RISI0294, 17 September 2020).

### 2.2. Serum Cyfra 21.1 and Thyroglobulin (Tg) Measurements

Serum concentrations of Cyfra 21.1 were measured using a specific immunoradiometric assay (IRMA, ELISA-CYFRA, Cisbio Bioassay, Codolet, France), in which Cyfra 21.1 molecules are sandwiched between the two mouse monoclonal antibodies KS 19.1 and BM 19.21 [17,18]. Excess unbound tracer was easily removed during the washing step, and the ELISA retained only the absorbed antibody/antigen/tracer antibody combination. The degree of radioactivity bound to the ELISA is proportional to the Cyfra 21.1 level present at the beginning of the assay. The reference range of Cyfra 21.1 was 0.0–3.6 ng/mL. Levels of Tg antibody (Tg antibody IRMA, Diasource, Louvain, Belgium) and Tg antigen (Tg IRMA, CIS Bio International, Gif sur Yvette, France) were also measured via immunoradiometric assays.

### 2.3. Pathologic Review

An experienced board-certified pathologist reviewed the tissues of DM-TC patients. Accordingly, the patients were subdivided into papillary thyroid cancer (PTC), follicular thyroid cancer (FTC), papillary + follicular type thyroid cancer (PTC + FTC), poorly differentiated thyroid cancer (PDTC) and anaplastic thyroid cancer (ATC) according to their pathology subtype.

### 2.4. ^131^Iodine Therapy Refractoriness

The effect of radioiodine therapy on every patient in the DM-TC group who underwent radioiodine therapy was reassessed. An experienced, board-certified nuclear medicine specialist reviewed every patient’s scan and compared with available morphological images. DM-TC patients were classified into three groups: (1) radioiodine-avid; (2) radioiodine-refractory; (3) unknown. The nuclear physician was unaware of the serum Cyfra 21.1 level or any other laboratory finding of the patient at the time of the classification. We defined radioiodine-refractory thyroid cancer according to the 2015 ATA guidelines as follows:

1. Absence of radioiodine uptake by malignant/metastatic tissue outside the thyroid bed during the initial therapeutic whole-body scan;
2. Loss of ability to concentrate radioiodine in the tumor tissue after previous evidence of radioiodine-avid disease (in the absence of stable iodine contamination);
3. Concentration of radioiodine in some lesions but not in others;
4. Progressive metastatic disease despite significant concentration of radioiodine [19].

### 2.5. BRAF Mutation

Genomic DNA was extracted from two 10-μm-thick paraffin sections containing a representative portion of each archival tissue block using the QIAamp DNA Mini kit (Qiagen, Hilden, Germany). The descriptions of the mutations were assigned according to “Guidelines for mutation nomenclature” from Human Genome Variation Society [20]. For B-Raf Proto-Oncogene, Serine/Threonine Kinase (BRAF)V600E mutation analysis, NBCI reference sequences-NG_007873.1 [21] and NM_004333.4 [22] were used 

### 2.6. One-Year Follow-Up of Serum Cyfra 21.1 Levels

The serum Cyfra 21.1 and serum Tg levels of 19 patients were followed up at one-year intervals. Several patients (*n* = 4) who had elevated Cyfra 21.1 levels at one-year follow-up underwent another one-year serial follow-up. Their disease status was evaluated using computed tomography (CT), bone scan, and positron emission tomography/CT (PET/ CT). The Response Evaluation Criteria in Solid Tumors (RECIST) (criteria were used to evaluate response to ablative therapies. Ablative therapies included external beam radiation or radiofrequency ablation, recurrent lymph node excision, radioiodine therapy and tyrosine kinase inhibitors [23].

The RECIST criteria were:

1. Complete response defined by disappearance of all target lesions;
2. Partial response defined by decrease in the number of metastatic nodules or 30% decrease in the sum of the longest diameter of target lesions;
3. Progressive disease involving increased number of metastatic nodules or 20% increase in the sum of the longest diameter of target lesions;
4. Stable disease with neither progression nor regression.

### 2.7. Statistical Analysis

Continuous variables were expressed as mean ± standard deviation or percentage unless otherwise stated. Categorical variables were described based on relative frequencies. For comparison, Student’s *t*-test or ANOVA was used for continuous variables with normal distribution. The Mann–Whitney or Kruskal–Wallis test was used to evaluate the differences between variables with non-normal distributions. The changes in variables before and after the time interval were compared. A two-tailed *p*-value less than 0.05 was statistically significant. All statistical analyses were performed using IBM SPSS Statistics for Windows v24.0 (IBM Corp., Armonk, NY, USA). Graphpad Prism V.7.00 software (GraphPad Software, La Jolla, CA, USA) was used to draw figures.

## 3. Results

### 3.1. Baseline Clinical Characteristics of Study Subjects and Their Serum Cyfra 21.1 Levels

**Figure 1:**
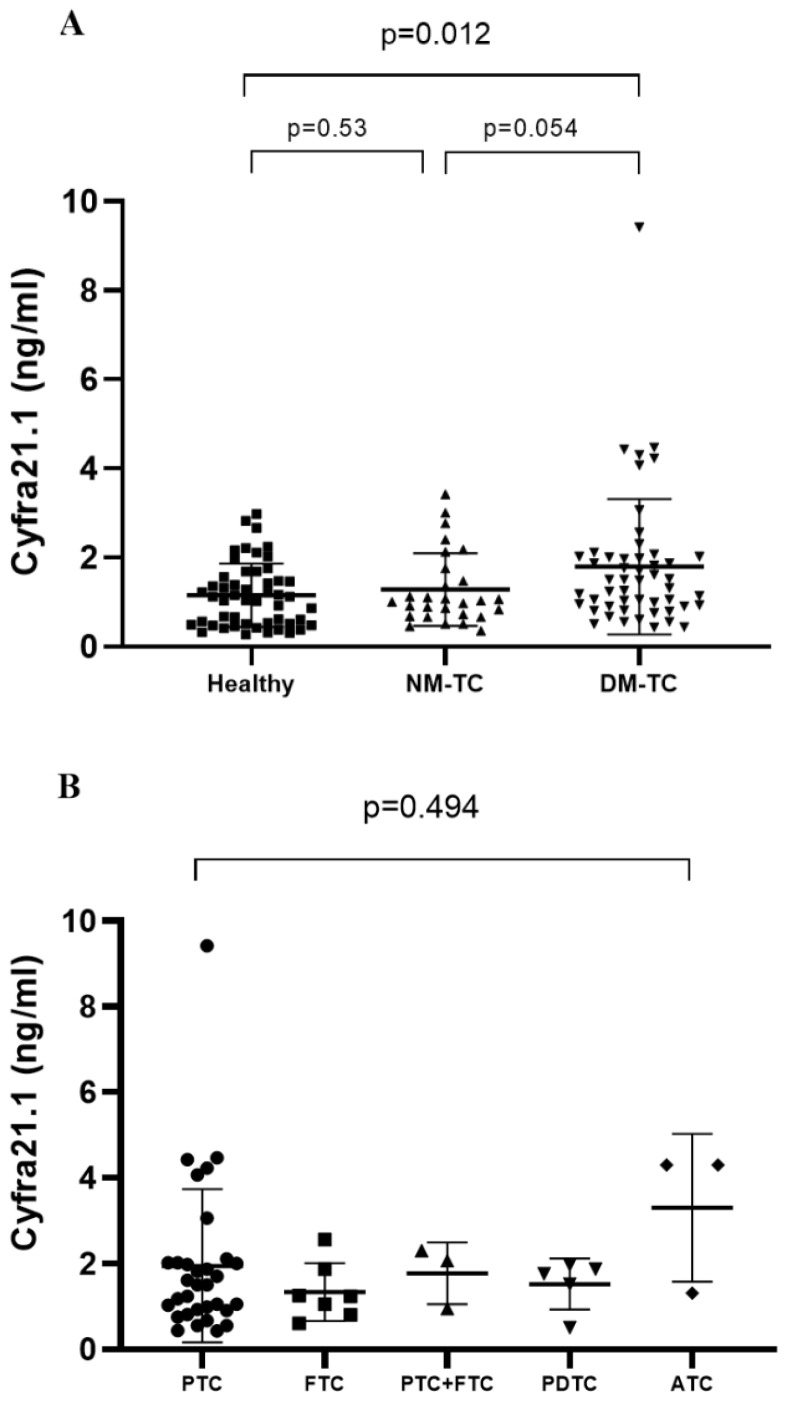
Serum Cyfra 21.1 levels in thyroid cancer according to study population (**A**) and histological subtype (**B**). PTC, papillary thyroid cancer; FTC, follicular thyroid cancer; PDTC, poorly differentiated thyroid cancer; ATC, anaplastic thyroid cancer; Healthy, healthy individuals with normal thyroid gland; NM-TC, thyroid cancer without distant metastasis; DM-TC, thyroid cancer with distant metastasis.

The baseline clinical characteristics of DM-TC are shown in Table 1. Serum Cyfra 21.1 levels of DM-TC were significantly higher than in Healthy groups but not in NM-TC (1.86 ± 1.58 ng/mL vs. 1.15 ± 0.71 ng/mL, *p* = 0.008; 1.86 ± 1.58 ng/mL vs. 1.30 ± 0.85 ng/mL, *p* = 0.054) (Figure 1A). We calculated the receiver operator characteristic (ROC) curve to know the cut-off level of Cyfra 21.1 as a diagnostic test looking for distant metastases in thyroid cancer (figure not shown). The cut-off level was 1.13 ng/mL (AUC = 0.631) with a sensitivity of 0.62 and specificity of 0.65. Serum Cyfra 21.1 levels in DM-TC patients were not associated with serum Tg levels (Spearman Coefficient 0.12) or with serum anti-Tg Ab level (Spearman Coefficient −0.2).

Thyroid cancer with distant metastasis was subdivided according to histological subtype (Figure 1B). The mean level of serum Cyfra 21.1 increased in ATC compared with PTC, FTC, PTC + FTC and PDTC but not statistically significantly, probably due to low numbers (*n* = 3) (*p* = 0.49).

### 3.2. Subgroup Analysis of Serum Cyfra 21.1 in Thyroid Cancer Patients with Distant Metastasis (DM-TC) Based on the Metastatic Site, Braf Mutation Status, Radioiodine Refractoriness, and Treatment with Tyrosine Kinase Inhibitor

**Figure 2:**
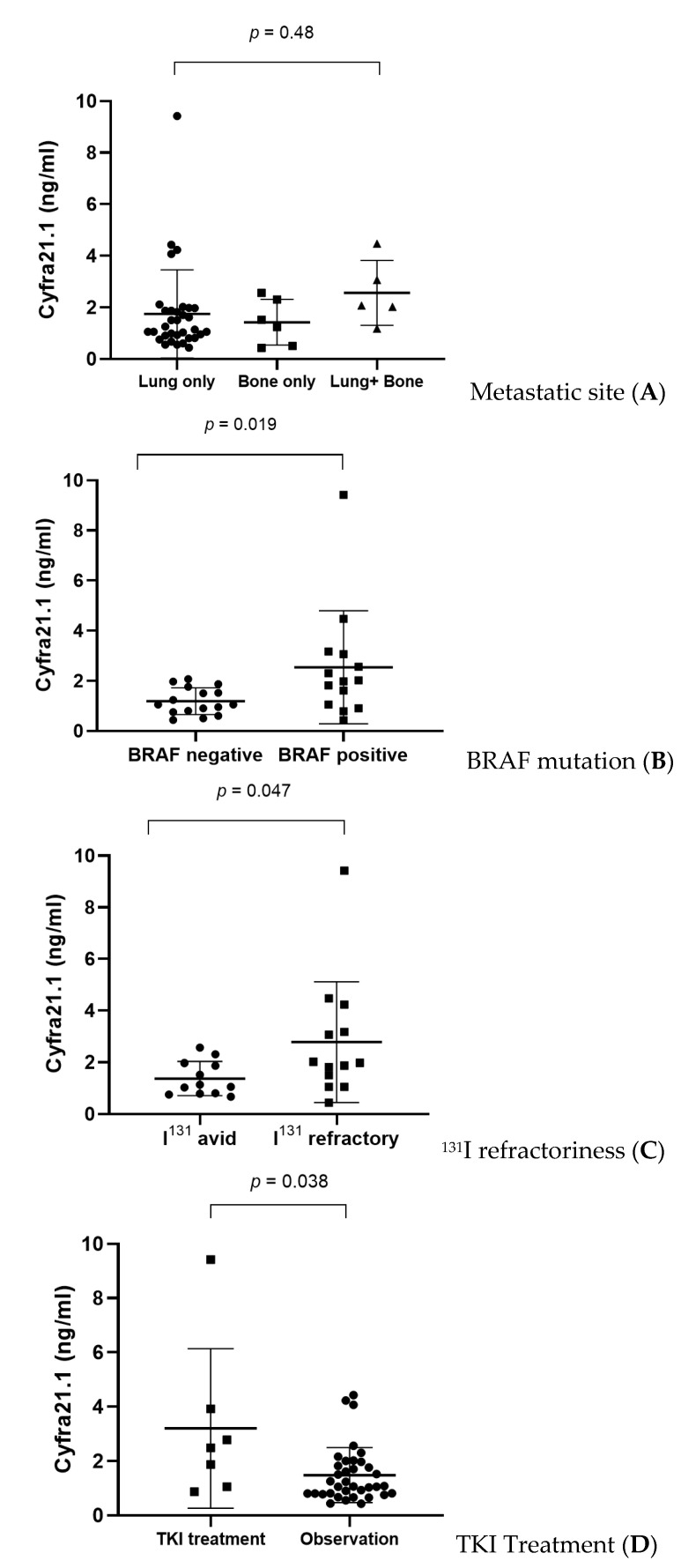
Subgroup analysis of serum Cyfra 21.1 levels in patients with thyroid cancer with distant metastasis (DM-TC) according to the metastatic site (**A**), BRAF mutation status (**B**), radioiodine refractoriness (**C**), and tyrosine kinase inhibitor (TKI) use (**D**).

The serum Cyfra 21.1 level of patients with concurrent lung and bone metastasis was higher than that of patients with metastasis involving either lung or bone alone, but without significance (*p* = 0.48) (Figure 2A). In the DM-TC group, BRAF-positive patients carried a higher level of serum Cyfra 21.1 than those testing negative for BRAF mutation. (2.01 ± 1.13 vs. 1.14 ± 0.52, *p* = 0.019) (Figure 2B). Among the 25 patients (49.0%) classified according to the radioiodine avidity of metastatic lesions, the serum Cyfra 21.1 level in the radioiodine-refractory group (*n* = 13) was significantly higher than in the radioiodine-avid group (*n* = 12) (*p* = 0.047) (Figure 2C). Seven patients treated with TKIs (2 with sorafenib and 5 with lenvatinib) showed significantly higher Cyfra 21.1 levels (3.20 ± 2.93) than patients without exposure to TKI (1.48 ± 1.01) (*p* = 0.038).

### 3.3. Serial Follow-Up of Serum Cyfra 21.1 Level

**Figure 3:**
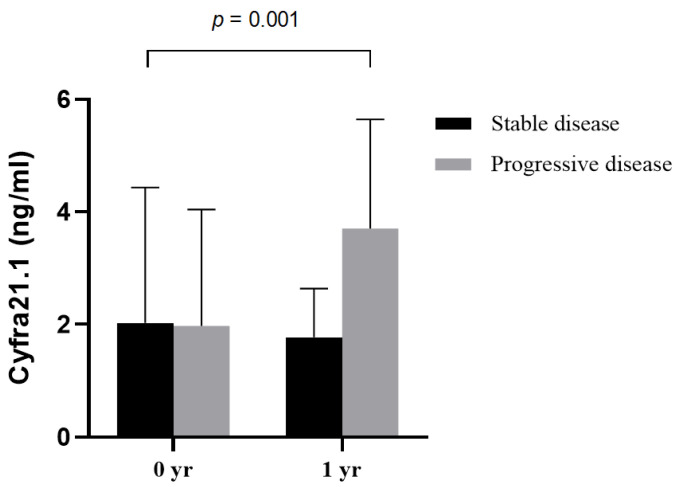
Changes in serum Cyfra 21.1 over one year of follow-up in patients with progressive disease (*n* = 6) and stable disease (*n* = 13);

The characteristics of 19 patients with one-year follow-up Cyfra 21.1 data are listed in Table 2. The levels of Cyfra 21.1 were higher in patients with progressive disease (*n* = 6) than in patients with stable disease (*n* = 13) (*p* = 0.007) (Figure 3).

During the one-year interval, seven patients were treated with TKI (sorafenib or lenvatinib). Among the seven patients, only five showed increased serum Cyfra 21.1 at one-year follow-up. Local ablative therapies (radiotherapy or recurrent lymph node excision) were administered to three patients (patient No. 4, 11 and 13). Local ablative therapies alone without TKI treatment did not alter Cyfra 21.1 level, although the number was too small to show significance.

### 3.4. Representative Patients with Serum Cyfra 21.1 as a Prognostic Biomarker for Disease Progression in Thyroid Cancer

**Figure 4:**
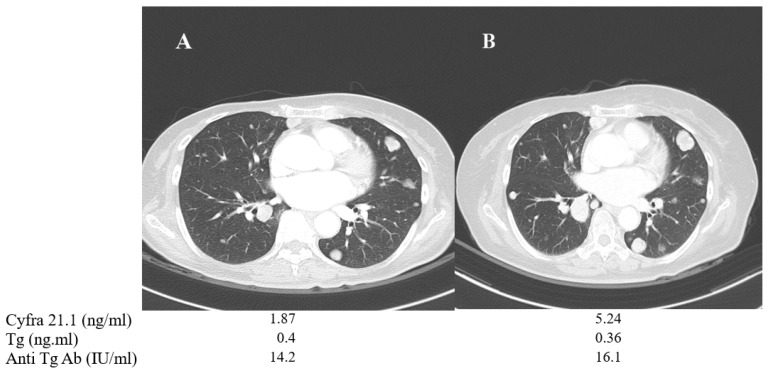
Changes in chest CT images and serum biomarkers of a 57-year-old female with poorly differentiated thyroid cancer (patient No. 1) before (**A**) and after one year of TKI treatment (**B**).

A 57-year-old female (patient No. 1) who had PDTC with negative BRAF mutation showed no detectable Tg or anti-Tg antibody levels, despite multiple lung metastases. Tg or anti-TgAb measurement is useless in verifying the disease progression in these cases of Tg non-secretary PDTC. Therefore, serum Cyfra 21.1 was used as an alternative tumor marker. It was monitored during disease progression under lenvatinib therapy. Serum Cyfra 21.1 was increased from 1.87 to 5.24 ng/mL when the total tumor size of three metastatic lung nodules showed a 33% increase based on RECIST criteria, while the serum Tg and anti-TgAb levels showed minimal change (Figure 4).

**Figure 5:**
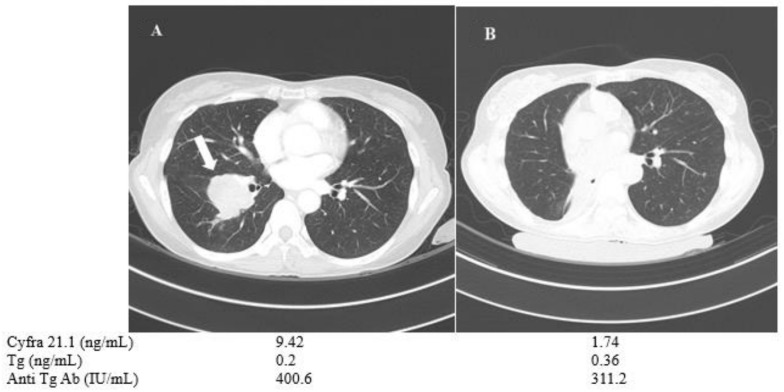
Change in chest CT images and serum biomarkers of a 50-year-old PTC female with Anti-Tg Ab (patient No. 8). The patient’s 4.6 cm tumor mass (arrow, **A**) was removed via bilobectomy of right middle and right low lobes (**B**).

A 50-year-old female (patient no. 8) who had advanced PTC with multiple lung metastases and a single large tumor mass (measuring 4.6 cm in maximal diameter) in the right lung showed low Tg and high anti-TgAb levels, which interfered with follow-up of the disease status based on Tg levels (Figure 5.) During the follow-up, the patient was treated with sorafenib, which resulted in stable disease. However, bilobectomy of right middle and low lobes was done to resect a slow-growing large tumor mass in the right lung, and thereby reduced the tumor burden in pulmonary metastasis. The post-operative pathology confirmed metastatic PTC, and next-generation sequencing revealed BRAF V600E mutation, TERT splicing mutation and EIF3E-RSPO2 gene fusion, indicating de-differentiation and poor prognosis. Surgery decreased the patient’s Cyfra 21.1 level remarkably, whereas Tg and anti-TgAb levels showed little or minor changes (Figure 5).

**Figure 6:**
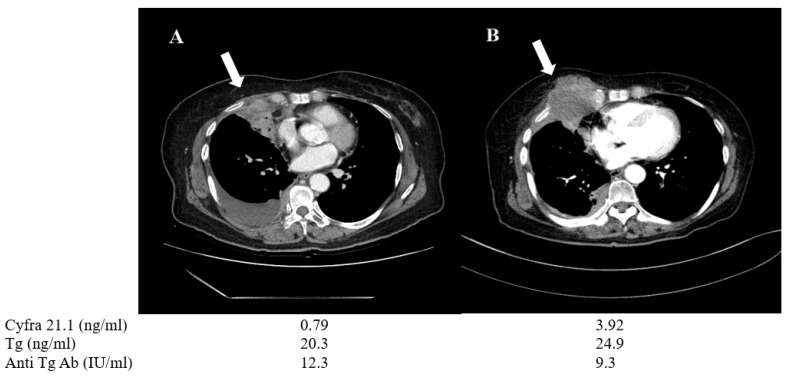
Changes in chest CT images and serum biomarkers of a 70-year-old PTC female (patient No. 6) before (**A**) and after one year of TKI treatment (**B**). During the time interval, the tumor progressed and changed to anaplastic feature (arrow).

A 70-year-old female patient (No. 6) was diagnosed with pleural wall and pulmonary metastasis derived from PTC during the initial presentation. She underwent radioiodine therapy with 150 mCi, but the metastatic lesions were refractory to treatment. The patient was treated with TKIs, including sorafenib and lenvatinib, sequentially for almost one year. During the treatment, her Cyfra 21.1 level increased from 0.79 to 3.92 ng/mL, while serum Tg level changed only from 20.3 to 24.9 ng/mL. Follow-up chest CT showed progressive disease involving chest wall tumor mass and the tissue biopsy confirmed anaplastic change from primary PTC, suggesting de-differentiation (Figure 6).

## 4. Discussion

Our study showed that serum Cyfra 21.1 level was correlated with distant metastasis and tumor progression of thyroid cancer. As tumor metastasis is a serial dynamic process involving the separation of tumor cells from the originating site, many tumor cells are destroyed during the metastatic cascade, releasing the structural component CK19 into the circulation [12]. Similarly, a previous study showed that serum Cyfra 21.1 levels obtained from fine-needle aspiration washout were significantly increased in metastatic lymph node in thyroid cancer [24]. We postulate that at the time of metastasis, the cleavage of CK19 by caspase 3 resulted in increased synthesis and elevation in serum levels of Cyfra 21.1. Similarly, during metastasis, proteolysis of keratin filaments or apoptosis leads to an increase in serum Cyfra 21.1 [25].

In one previous study, Gao et al. [15] reported that serum Cyfra 21.1 in thyroid cancer patients did not show any differences between those with pulmonary and without distant metastasis. However, Gao et al. used therapeutic whole-body scan (TxWBS) to distinguish patients with pulmonary metastasis from those without distant metastasis. Therefore, patients with multiple pulmonary metastasis who showed no uptake in TxWBS (suggesting de-differentiation and radioiodine refractoriness) and high serum Cyfra 21.1 level may have been misdiagnosed as absence of distant metastasis. In our study using CT, PET/CT, and TxWBS to diagnose distant metastasis, DM-TC showed higher serum Cyfra 21.1 levels than NM-TC, but this was statistically insignificant (*p* value = 0.054). Therefore, the potential utility of serum Cyfra 21.1 as a diagnostic test looking for distant metastasis in thyroid cancer was limited, considering the low sensitivity and specificity with ROC curve analysis. We speculate that since serum Cyfra 21.1 is elevated mainly in ^131^I-refractory thyroid cancer patients or BRAF-mutation-positive patients, using serum Cyfra 21.1 as a diagnostic test of distant metastasis in ^131^I-avid thyroid cancer patients or BRAF-mutation-negative patients might lower the accuracy. Further study is needed to clarify the issue.

Unlike the previous study of Giovanella et al. [26], which showed higher serum Cyfra 21.1 levels in PDTC and ATC, the PDTC in our study showed similar Cyfra 21.1 levels to those of DTC, probably due to the small number of PDTCs, and a proportion of PDTC may grow at a pace similar to other DTCs, like FTC or PTC.

It is known that the BRAF V600E mutation, which is the most common in DTC, is associated with a higher percentage of CK19-expressing cells [27]. BRAF V600E mutation is a key driver of PTC and is associated with a more aggressive behavior [28]. Lately, Wang et al. [29] reported that in thyroid cancer cells with positive BRAF V600E mutation, increased CK19 expression might promote epithelial–mesenchymal transition (EMT) characterized by decreased E-cadherin and increased N-cadherin expression, resulting in lymph node metastasis. Accordingly, we found that serum Cyfra 21.1, the soluble fragment of CK19, was increased in thyroid cancer patients with distant metastasis and BRAF V600E mutation, indicating that serum Cyfra 21.1 levels in this group may facilitate disease monitoring; however, further studies are needed to validate this finding. As seen in the case of a 50-year-old female (patient No. 8), multiple driver mutations (BRAF V600E mutation, TERT splicing mutation and EIF3E-RSPO2 gene fusion) affect CK19 expression and increase serum Cyfra 21.1 levels; however, it is still unknown whether specific driver mutations (except BRAF V600E) may further increase serum Cyfra 21.1 levels.

In malignant epithelial cells, activated protease increases the degradation of CK19 and releases CK19 fragments into the blood stream, including a water-soluble fragment Cyfra 21.1 [12]. In previous studies, caspase 3, which cleaves several intermediate filaments and mediates cellular apoptosis, played an important role in producing Cyfra 21.1 [30]. Caspase 3 is known not only to increase the sensitivity of cancer cells to chemotherapy and radiotherapy, but also to induce cancer cell invasion and metastasis [31]. Tumor metastasis entails a complex interplay of stem cell renewal, differentiation and apoptosis. Caspase 3 is involved in multiple steps in the stem cell life-cycle, affecting development and differentiation [32]. In a study evaluating the expression of caspase 3 in metastatic lymph nodes, a higher level of caspase 3 expression was observed in metastatic lymph nodes than in primary tumors in esophageal squamous cell carcinoma [33]. Therefore, the activity of caspase 3 appears to increase further in metastatic tissue and higher caspase 3 expression in multiple metastatic tumors may result in higher serum Cyfra 21.1 levels. Accordingly, we can speculate that thyroid cancers with multiple distant metastases might show higher serum Cyfra 21.1 levels compared with those without distant metastasis, although we were unable to measure caspase 3 activity in metastatic lesions.

In thyroid cancer patients with distant metastasis, the ability to concentrate radioiodine and to synthesize and secrete Tg is decreased (tumor cell de-differentiation), leading to poor response to radioiodine therapy and difficulty in monitoring the disease based on serum Tg level [34,35]. ^131^I-refractory thyroid cancer cells [36] are likely another source of increased serum Cyfra 21.1 [37].

Tg is the most traditional biomarker in the follow-up of DTC. However, as seen above, serum Tg cannot be used as a biomarker for disease monitoring in several clinical cases and it is unknown whether serum Cyfra 21.1 level could be used as an alternative option. Campenni et al. reported that low postsurgical Tg value do not rule out metastases in early stage of DTC [38]. When advanced thyroid cancer patients with multiple metastasis do not secrete Tg or Tg is undetectable because of anti-TgAb, serial follow-up based on serum Cyfra 21.1 levels might be used as an alternative biomarker for disease monitoring comparable to serum Tg, especially in BRAF-mutated thyroid cancer.

However, there is a caveat to interpreting serum Cyfra 21.1 in advanced thyroid cancer treated with TKI. As TKI may induce apoptosis [39] and increase serum Cyfra 21.1, it is better to predict disease course in de-differentiated and metastatic thyroid cancer by measuring baseline serum Cyfra 21.1 before using TKI and at the follow-up during the disease progression. Given that patients who need TKI therapy usually manifest aggressive growth and radioiodine-refractory thyroid cancer, we cannot establish whether the increased Cyfra 21.1 levels of these patients are derived from progressive tumor or TKI therapy.

Previous reports demonstrated that Cyfra 21.1 was associated with a high degree of malignancy and aggressive DTC biology [14]. Low levels of Cyfra 21.1 are associated with favorable outcome [16]. Apparently, the mechanism is still uncertain but a high level of Cyfra 21.1 showed de-differentiation leading to poor response to conventional therapy, and also promoted metastasis via EMT. Our data established an alternative role of serum Cyfra 21.1 as a de-differentiation biomarker instead of serum Tg, which is a marker of differentiated thyroid cancer [16].

The study has several limitations. First, the small number of subjects does not validate the results, although the study population predominantly involves advanced and metastatic thyroid cancer. Therefore, further studies with large cohorts are necessary to corroborate our results. Second, it is known that cytokeratin expression differs from PTC subtypes. It is reported that follicular variant PTC (best prognosis) exhibited the least CK19 expression, while the tall cell variant PTC (worst prognosis) showed the highest CK19 expression [29]. Serum Cyfra 21.1 level might also differ according to PTC or FTC subtypes, although we were unable to distinguish them.

## 5. Conclusions

Serum Cyfra 21.1 levels were significantly increased in metastatic thyroid cancer patients with disease progression, compared with those manifesting stable disease. Serum Cyfra 21.1 level in thyroid cancer patients might be used as an alternative biomarker to predict tumor progression, especially in cases with undetectable or unmonitored thyroglobulin because of thyroglobulin antibody.

## Figures and Tables

**Table 1:** Baseline clinical characteristics of the study population.

Clinical Variables	Metastasis *n* = 51	No Metastasis *n* = 26	Healthy Controls *n* = 50	*p*-Value
Age (years)	53.6 ± 12.8	55.6 ± 9.0	58.2 ± 13.4	0.27
Gender (M/F)	19/32	15/11	14/36	0.04
Cyfra21.1 (ng/mL)	1.86 ± 1.58	1.30 ± 0.85	1.15 ± 0.71	0.01
Thyroglobulin (ng/mL)	258.85 ± 635.5	12.69 ± 16.78	NA	0.02
Anti-thyroglobulin Ab (IU/mL)	48.49 ± 114.57	14.79 ± 15.84	NA	0.03
Subtypes				
Papillary	33	25	-	-
Follicular	7	1	-	-
Papillary + Follicular	3	0	-	-
Poorly differentiated	5	0	-	-
Anaplastic	3	0	-	-
Distant metastasis site				
Lung only	40	-	-	-
Bone only	6	-	-	-
Both lung and bone	5	-	-	-
BRAF mutation				
Positive	16	-	-	-
Negative	23	-	-	-
Unknown	12	-	-	-
Response to radioiodine therapy				
Avid	12	-	-	-
Refractory	13	-	-	-
Unknown	26	-	-	-

Continuous variables are presented as mean ± standard deviation; Categorical variables are presented as number (percentage); NA, not available.

**Table 2:** One-year follow-up data of serum Cyfra 21.1 and thyroglobulin levels in thyroid cancer with distant metastasis (DM-TC) group.

No.	Sex	Age	Subtype	Initial Tg (TgAb)	1 Year Follow-Up Tg (TgAb)	Initial Cyfra 21.1	1 Year Follow-Up Cyfra 21.1	Metastasis	State	Treatment
1	F	57	PDTC	0.4 (14.2)	0.36 (16.1)	1.87	5.24	Lung	Progression	TKI (LE)
2	F	60	FTC	741	1671.4	1.05	2.16	Lung	Progression	Observation
3	F	74	PTC	28.0	68.9	3.06	6.32	Lung	Progression	Observation
4	F	62	PTC	98.4	33.1	0.9	2.49	Lung	Progression	TKI * (SO)/LN excision
5	M	65	PTC	140.7	183.8	1.98	2.3	Lung	Progression	Observation
6	F	70	PTC > ATC	20.3	24.9	0.79	3.92	Lung	Progression/ Change to anaplastic feature	TKI*(LE + SO)
7	F	59	PTC + FTC	2519	964	2.07	0.87	Lung + Bone	Stable	TKI * (LE)
8	F	50	PTC	0.2 (400.6)	0.2 (333.9)	9.42	7.61	Lung	Stable	TKI (SO)
9	F	60	PTC + FTC	58.0	0.36	0.95	2.78	Lung	Stable	TKI * (L E)
10	M	62	PTC	11.3	17.6	1.87	0.78	Lung	Stable	Observation
11	F	57	PTC	4.1	3.2	0.6	0.81	Lung	Stable	Observation/LN excision
12	F	55	PTC	21.9	32.7	1.14	1.06	Lung	Stable	Observation
13	M	55	PDTC	58.8	2.6	0.5	0.66	Bone	Stable	Resection of metastatic site/RT
14	M	62	PTC + FTC	19.2	39.9	2.3	1.08	Bone	Stable	RAI
15	F	55	PTC	0.88 (495.2)	0.36 (246.0)	1.5	0.8	Lung	Stable	Observation
16	M	62	PTC	1.8	2.0	2.11	0.65	Lung	Stable	Observation
17	F	78	PTC	86.6	37.3	1.05	2.65	Lung	Stable	TKI (LE)
18	F	73	PTC	34.6	35.7	1.61	0.91	Lung	Stable	Observation
19	F	55	PTC	20.5	20.2	0.99	0.52	Lung	Stable	Observation

Tg, Thyroglobulin; TKI, tyrosine kinase inhibitor; RAI, radioactive iodine therapy; SO, sorafenib; LE, lenvatinib; RT, radiotherapy. * TKI started in the middle of interval follow-up.

## Data Availability

Not applicable.
